# A commentary on “Transanal Opening of Intersphincteric Space (TROPIS) treatment for high complex anal fistula: a systematic review and meta-analysis”

**DOI:** 10.1097/JS9.0000000000003198

**Published:** 2025-08-21

**Authors:** Xiaolian Deng, Huayang Pang, Chunxue Li

**Affiliations:** a Department of General Surgery, Daping Hospital, Army Medical University (Third Military Medical University), Chongqing, China; b Department of Gastrointestinal Cancer Center, Chongqing University Cancer Hospital, Chongqing, China

*Dear editor,*

We read with great interest the study conducted by Zhou *et al*^[1]^, which investigated the efficacy and safety of transanal opening of intersphincteric space (TROPIS) in the management of patients with highly complex anal fistula. This meta-analysis, encompassing six studies involving 499 patients, revealed that TROPIS is a secure and effective procedure for such patients. It exhibited a high one-time cure rate (80%), a low recurrence rate (20%), a high overall cure rate (89%), and no adverse events were reported. The authors deserve acclaim for presenting evidence that supports the superiority of TROPIS in highly complex anal fistula management. Nevertheless, several aspects require further elucidation prior to translating these findings into clinical practice.

A comprehensive literature search is an essential prerequisite for guaranteeing the reliability of a meta-analysis. In this particular study, the authors solely conducted searches in PubMed, LISTA, and Web of Science to identify potential literature. Nevertheless, several other frequently utilized databases, such as Embase and the Cochrane Library, were not explored by the authors. Herein, we identified a significant study by Reyes Díaz *et al*^[2]^ that was published on 17 December 2024 and was overlooked by the authors. After a meticulous evaluation of its full text, we ascertained that this study indeed met the inclusion criteria for this meta-analysis. Consequently, it would have been appropriate for the authors to incorporate it into their analysis.

Secondly, during the assessment of the characteristics of the included studies, it was noted that two studies^[3,4]^ originated from the same center. Specifically, one study^[3]^ recruited patients from 2015 to 2016, whereas the other^[4]^ enrolled patients between 2015 and 2020. Consequently, a substantial overlap exists among the patients included in these two studies. The repeated inclusion of the same patients clearly contravenes the principles of meta-analysis. Hence, we propose that the authors meticulously evaluate the rationality of the included literature and retain only the appropriate one for analysis.

Overall, we express our sincere gratitude to Zhou and colleagues for their collaborative and dedicated efforts in exploring the clinical value of TROPIS in patients with highly complex anal fistula. Their work represents a significant advancement in this clinically relevant area. However, we would like to raise some concerns, which may potentially help the authors improve the clarity of their research findings.

This study is compliant with the TITAN Guidelines 2025^[5]^.

## Data Availability

No data used in this letter to the editor.

## Research registration unique identifying number (UIN)

No need for this letter to the editor.
